# Antimicrobial peptides CS-piscidin-induced cell death involves activation of RIPK1/PARP, and modification with myristic acid enhances its stability and tumor-targeting capability

**DOI:** 10.1007/s12672-023-00642-1

**Published:** 2023-03-31

**Authors:** Ning Li, Xingmei Jiang, Xiaowan Ma, Xiaoju Qiu, HuangHuang Chang, Ying Qiao, Hui Luo, Qingyu Zhang

**Affiliations:** 1 Laboratory of Obstetrics and Gynecology, Department of Obstetrics and Gynecology, Affiliated Hospital of Guangdong Medical University, Zhanjiang, 524001 Guangdong China; 2 The Marine Biomedical Research Institute of Guangdong Zhanjiang, Guangdong Medical University, Zhanjiang, 524023 China; 3 Department of Hematology, Affiliated Hospital of Guangdong Medical University, Zhanjiang, 524001 Guangdong China; 4 Key Laboratory of Tropical Marine Ecosystem and Bioresource, Fourth Institute of Oceanography, Ministry of Natural Resources, Beihai, 536000 China

**Keywords:** Anti-cancer peptide, Peptide myristylation modification, Ovarian cancer

## Abstract

Ovarian cancer (OC) is a highly lethal gynecological malignancy, often diagnosed at advanced stages with limited treatment options. Here, we demonstrate that the antimicrobial peptide CS-piscidin significantly inhibits OC cell proliferation, colony formation, and induces cell death. Mechanistically, CS-piscidin causes cell necrosis by compromising the cell membrane. Furthermore, CS-piscidin can activate Receptor-interacting protein kinase 1 (RIPK1) and induce cell apoptosis by cleavage of PARP. To improve tumor targeting ability, we modified CS-piscidin by adding a short cyclic peptide, cyclo-RGDfk, to the C-terminus (CS-RGD) and a myristate to the N-terminus (Myr-CS-RGD). Our results show that while CS-RGD exhibits stronger anti-cancer activity than CS-piscidin, it also causes increased cytotoxicity. In contrast, Myr-CS-RGD significantly improves drug specificity by reducing CS-RGD toxicity in normal cells while retaining comparable antitumor activity by increasing peptide stability. In a syngeneic mouse tumor model, Myr-CS-RGD demonstrated superior anti-tumor activity compared to CS-piscidin and CS-RGD. Our findings suggest that CS-piscidin can suppress ovarian cancer via multiple cell death forms and that myristoylation modification is a promising strategy to enhance anti-cancer peptide performance.

**Graphical Abstract:**

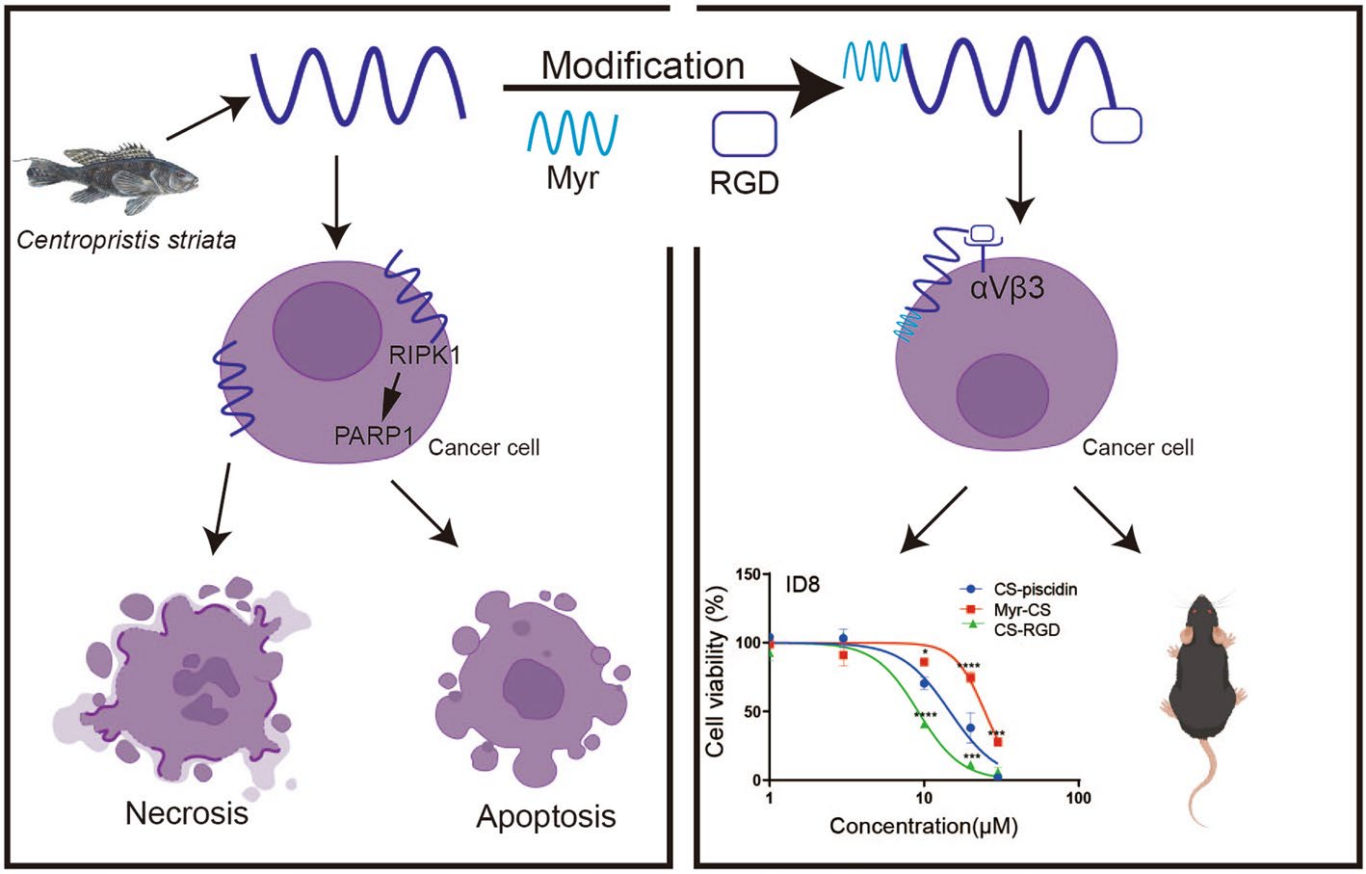

**Supplementary Information:**

The online version contains supplementary material available at 10.1007/s12672-023-00642-1.

## Introduction

Ovarian cancer is one of the most common malignancies tumors worldwide [1]. More than 65% of patients lost their lives within 5 years of diagnosis [2]. Due to the absence of specific symptoms and reliable screening methods, OC is often challenging to diagnose until it has metastasized to the omentum, peritoneum or other distant organs [3]. Neoadjuvant chemotherapy followed by debunking surgery is the conventional treatment approach of ovarian cancer. Afterwards, combination of carboplatin with paclitaxel or bevacizumab was used to help prevent cancer relapse [4]. Unfortunately, the persistent use of small molecular drug often leads to drug resistance, posing a significant obstacle that needs to be overcome.

Antimicrobial peptides (AMPs) are natural peptides existed in the organism which provide innate immunity to protect them from microbial infection. Tumors arise from the malignant transformation as mutation accumulation in normal cells. AMPs have been reported to have versatile functions in medical applications, for example, anti-infection, tissue regeneration and cancer elimination. Previously, we isolated one peptide (20Aa) from the skin of *Centropristis striata,* which had strong antimicrobial ability in several tested bacteria [5]. Piscidin is a class of antimicrobial peptides originally identified in fish, thus we named this peptide CS-piscidin. Cyclization [6], methylation [7] and myristylation [8] of peptide can improve the physical and chemical properties to prolong half-life time or enhance bioavailability. Myristic acid is a 14:C fatty acid, which is more hydrophilic than palm acid (16:C) or stearic acid (18:C) and has been found to be less cytotoxic. Addition of myristic acid to peptides has been shown to improve their stability and lipophilic properties [8]. In addition, arginine-glycine-aspartate (RGD) peptide has been widely recognized for its ability to bind to integrins αvβ3 [9], which is typically high expressed in tumor cells [10]. It is commonly used to enhance the tumor targeting of liposomes and nanomaterials in cancer cells [11–14]. A cyclic RGDfk tumor-targeting peptide that resists enzyme digest had been found to have a longer half-life and better tumor targeting than linear peptide [15, 16].

RIPK1 is a member of the receptor-interacting protein (RIP) family of serine/threonine kinases, which regulates cellular processes such as cell death, inflammation, and immune responses [17]. PARP (poly ADP-ribose polymerase) is a crucial enzyme responsible for DNA single-strand break repair. When DNA is damaged, PARP recognizes the damage and recruits DNA-repair effectors to initiate the repair process [18]. Cell death complex IIa formation and RIPK1 phosphorylation trigger apoptosis, which is activated by caspase 8 cleaving of PARP [19]. If caspase 8 is inactive, necroptosis is initiated by RIPK1 and RIPK3 activation to form a necrosome. The necrosome activates MLKL, which punctures the cell membrane, releasing intracellular contents and resulting in cell necroptosis [19]. CS-piscidin is an amphipathic peptide that primarily induces cell death by disrupting cell membranes. While it causes rapid cell necrosis, it also gradually induces cell death, suggesting the involvement of other regulatory cell death processes.

Here, we investigated the anti-tumor effect of the marine-derived AMPs CS-piscidin in ovarian cancer. We also modified the peptide by adding a cyclo-RGD and myristate group. Results showed that myristoylation of CS-piscidin improved its tumor targeting and stability, indicating that this modification is a viable strategy to improve the performance of antitumor peptides.

## Materials and methods

### Peptides and modification

The structure of the peptide and the modified peptide are presented in Table 1:

**Table 1:** The sequence and structure of the peptides

Name	Molecule chemistry structure
CS-piscidin	
CS-RGD	
Myr-CS-RGD	

These peptides were synthesized by Qiangyao Biotechnology Co., Ltd (Shanghai, China) with a purity > 95%. The peptides were dissolved in autoclaved ultrapure water.

### Reagents and antibodies

The following reagents were used in this study: DMEM (Gibco, 8,121,334, USA), Fetal bovine serum (Biological Industries, 04-001-1ACS, Israel), penicillin–streptomycin liquid (Solarbio, P1400, China), trypsin (Solarbio, T1350, China), RIPA Lysis Buffer (Beyotime, P0013B, China), BCA Protein Assay Kit (Beyotime, P0012, China), anti-Tubulin (1:1000, CST, 2125, USA), anti-PARP (1:1000, CST, 9532, USA), anti-p-RIPK1 (1:1000, CST, 65,746, USA), anti-RIPK1 (1:1000, CST, 3493S, USA), Anti-Integrin β3(1:1000, Beyotime, AF1444, China), Pentobarbitone (Sigma, P3761, USA), Cell Counting Kit-8 (Beyotime, C0038, China), PL-luciferase (Addgene, 21,471, USA), Annexin V-FITC/PI Apoptosis Kit (Biosharp, BL110A, China), and Luciferin (Beyotime, ST196, China).

### Cell culture

The A2780, SKOV3, and OVCA429 human ovarian cancer cell lines, the ID8 mouse ovarian cancer cell line, the IOSE80 normal ovarian epithelial cell line, and the HUVEC human umbilical vein endothelial cell line were obtained from Shanghai Cell Bank and Professor Tsz on Lee University of Macau. The cell lines were cultured in DMEM supplemented with 10% certified fetal bovine serum and 1% penicillin–streptomycin and maintained in a humidified incubator at 37 °C with 5% CO2.

### Cell viability assay

A2780, SKOV3, ID8, IOSE80, and HUVEC cells (2.0 × 10^3^ per well) were seeded into 96-well plates in 100 μL culture medium. After 24 h of incubation, the medium was replaced with a medium containing various peptide concentrations. The cell viability after the peptide treatment was tested by CCK8 assay. Briefly, after 24 h treatment, 10 μL CCK8 was added to each well and incubated for 2 h. After incubation, the optical density (OD) at 450 nm was detected using a microplate reader. Cell viability was calculated using the formula: (%) = [D-D_0_]/[$\overline{\text{D}}$ -D_0_] × 100%. D was the OD value of the experimental group, D_0_ represented the OD value of the blank control group, while [$\overline{\text{D}}$ was the mean OD value of the control group.

### Colony formation assay

Logarithmically growing cells were trypsinized and 300 cells/well were seeded in a 6-well plate. After 10 h, the culture medium was replaced with medium containing peptides at IC50 and 2 × IC50 concentrations. The colonies were washed twice with PBS, fixed with 4% paraformaldehyde for 10 min, and stained with 0.1% crystal violet solution at room temperature for 15 min. The colonies were then photographed and counted using Image J software.

### Spheroids formation assay

The method can refer to previous studies [20, 21]. Briefly, the 6-well plate was pre-coated with 0.5% agarose gel, and 3000 cells were seeded in each well with medium containing a series of peptides. After 7–10 days of growth, the spheroids were transferred to normal plates to allow them to attach and grow for another 7–10 days. The colonies were then fixed and stained with crystal violet, as described in the colony formation assay.

### Apoptosis assay

The cells were seeded into 6-well plates and treated with fresh medium containing different concentrations of peptide for 24 h. A2780 cells were treated with 2, 4, and 8 μM while OVCA429 cells were treated with 10, 20, and 40 μM. After treatment, cells were harvested and counted. 1 × 10^5^ cells were used for each sample and resuspended in staining buffer. Then, 5 μL of PI and 5 μL of Annexin V-FITC were added and the samples were incubated for 10 min. The samples were then analyzed by flow cytometry (BD LSRFortessa™ X-20, BD Bioscience, USA). PI and Annexin V-FITC single staining samples were used for compensation adjustment.

### Western blotting analysis

A2780 and OVCA429 cells (3 × 10^5^ per well) were treated with various peptide concentrations for 24 h. After washing with PBS, cells were lysed with RIPA buffer and protein samples (20–30 μg) were loaded for gel separation and transferred to PVDF membranes. After blocking with 5% non-fat milk, membranes were incubated with primary antibodies (1:1000) at 4 ℃ overnight. HRP-conjugated secondary antibodies (1:5000) were used followed by detection with ECL substrate in an illuminance imaging system.

### Scanning Electron Microscope (SEM)

After cell attachment, cells were fixed in 2.5% glutaraldehyde for 2 h at room temperature. Tissue blocks were post-fixed in 1% OsO4, washed in 0.1 M PBS (pH 7.4), dehydrated in ethanol, treated with isoamyl acetate, and dried with a Critical Point Dryer. Specimens were then attached to metallic stubs, sputter-coated with gold, and imaged using a scanning electron microscope (SU8100, HITACHI).

### Construction of luciferase reporter cell line

To create luciferase heredity stable ID8 cells, the PL-luciferase plasmid (Addgene, 21,471) was packaged by co-transfecting it with DVPR and VSVG packing plasmids using PEI in HEK293T. The virus-containing solution was then collected and cell debris was removed through a 0.45 μm filter. After infection, the cells were selected by treating them with 1 μg/mL puromycin for 3 days to eliminate the uninfected ID8 cells. The expression of luciferase in the cells was then confirmed using the Bio-Lumi™ Firefly Luciferase Reporter Gene Assay Kit (RG042S, Beyotime, China).

### Ovarian cancer peritoneal spread mouse model

The animal experiment followed the Declaration of Helsinki guidelines and was approved by the Guangdong Medical University Animal Ethical Committee (GDY2102330). 5-week-old female C57BL/6 mice were obtained from Yancheng biological company and housed in an SPF laboratory at 26 ℃ with free access to water and food. Mice were intraperitoneally injected with 5 × 10^6^ ID8-Luc-puro cells resuspended in 200 μL PBS. On the seventh day after cell inoculation, mice were intraperitoneally administered with 5 mpk (mg/kg) tested peptides every two days for ten times, while PBS was given as a control. Body weight was measured every 5 days. After treatment, mice were injected with pentobarbitone and D-luciferin, and the luminescence signals were observed within 15 min using the live imaging system (*In-vivo* Xtreme, Bruker, USA). The fluorescence intensity was normalized and analyzed by Bruker MI SE software.

### Statistical analysis

At least three independent experiments were carried out and results were presented as mean ± SEM. All experimental data were statistically analyzed by using GraphPad 8.0. Two-tailed, unpaired Student’s t-test or ANOVA with Tukey post-hoc analysis was conducted to test the statistical significance of the data. For animal experiments, all animal studies were conducted using three to six animals per group for each experiment. At least two independent experiments were carried out and the statistical significance was determined using Student’s t-test. P < 0.05 was considered significantly statistical different. *Represented p < 0.05, **represented p < 0.01, ***represented p < 0.001.

## Results

### CS-piscidin inhibits human ovarian cell growth and increases carboplatin sensitivity

**Fig. 1:**
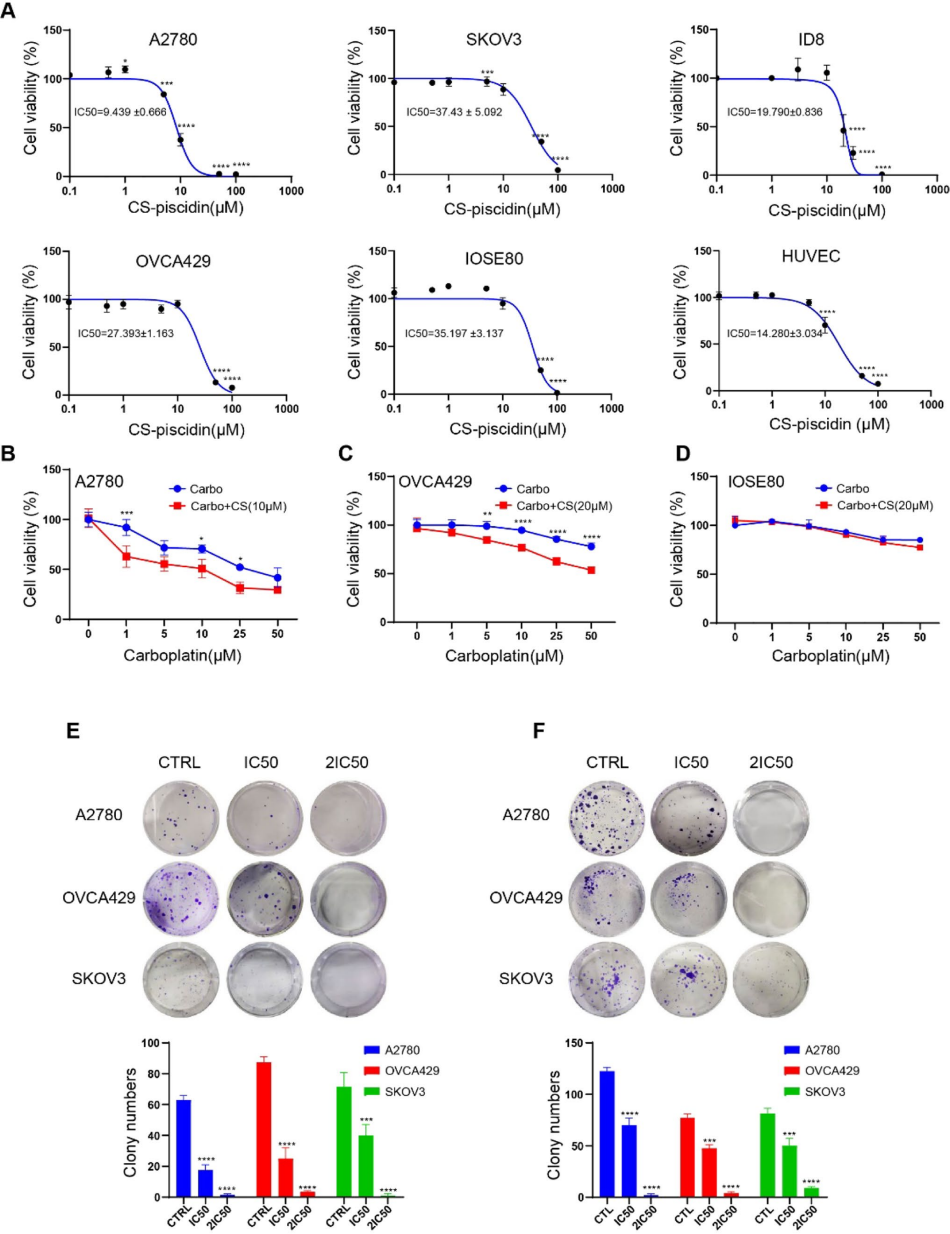
Antitumor ability and cytotoxicity of Cs-piscidin in vitro. **A** Cell viability of A2780, SKOV3, ID8, OVCA429, IOSE80, and HUVEC cells was determined using a CCK-8 assay after treatment with Cs-piscidin for 48 h. **B**–**D** The combination of Cs-piscidin with carboplatin was used to determine the inhibitory effect on the cell viability of A2780, OVCA429, and IOSE80 cells using a CCK-8 assay. **E** The inhibitory effect of Cs-piscidin on the single cell tumorigenic ability was measured by a colony formation assay in A2780, OVCA429, and SKOV3 cells. **F** The suppression effect of Cs-piscidin on spheroid formation in A2780, OVCA429, and SKOV3 cells was evaluated. Each data point represents the mean ± SEM of three independent experiments. ** p < 0.01, *** p < 0.001, and **** p < 0.0001 indicate statistical significance when compared to the control

Previously, our lab isolated and identified an AMPs peptide (FFKRLRNKFRL MRQAWKDYR) from skin mucus of *Centropristis striata* named CS-piscidin. To evaluate the inhibitory effect of CS-piscidin on OC cells, OC cells lines A2780, SKOV3, ID8 and OVCA429 were tested. Our results demonstrate that CS-piscidin reduces cell viability in a dose-dependent manner (Fig. 1A), with IC50 values of 9.439 ± 0.666 μM, 37.43 ± 5.092 μM, 19.790 ± 0.836 μM, and 27.393 ± 1.163 μM for cell lines A2780, SKOV3, ID8, and OVCA429, respectively. CS-piscidin also inhibits normal ovary epithelial cell IOSE80 with an IC50 of 35.197 ± 3.137 μM, which is higher than most tested ovarian cancer cell lines, except SKOV3. Notably, CS-piscidin can suppress human umbilical vein endothelial cells HUVEC with an IC50 of 14.280 ± 3.034 μM (Fig. 1A), suggesting it has anti-angiogenic potential. Carboplatin resistance is a significant clinical challenge, but the combination of carboplatin with other agents can achieve a synergistic effect. Combining CS-piscidin with carboplatin enhances the sensitivity of cancer cells to carboplatin (Fig. 1B–D). In colony formation assays, we found that CS-piscidin can inhibit colony formation of A2780, SKOV3, and OVCA429 cells in a dose-dependent manner (Fig. 4E). Ovarian cancer often metastasizes through the peritoneal cavity, where suspension cells can form multicellular aggregates called spheroids. To mimic ovarian cancer suspension and spheroid formation, we cultured cells in an ultralow attachment plate [22]. Our results demonstrated that treatment with CS-piscidin significantly reduces spheroid formation in A2780, SKOV3, and OVCA429 cell lines, confirming its antitumor ability (Fig. 1F).

### CS-Piscidin activates RIPK1 and induces ovarian cancer apoptosis

**Fig. 2:**
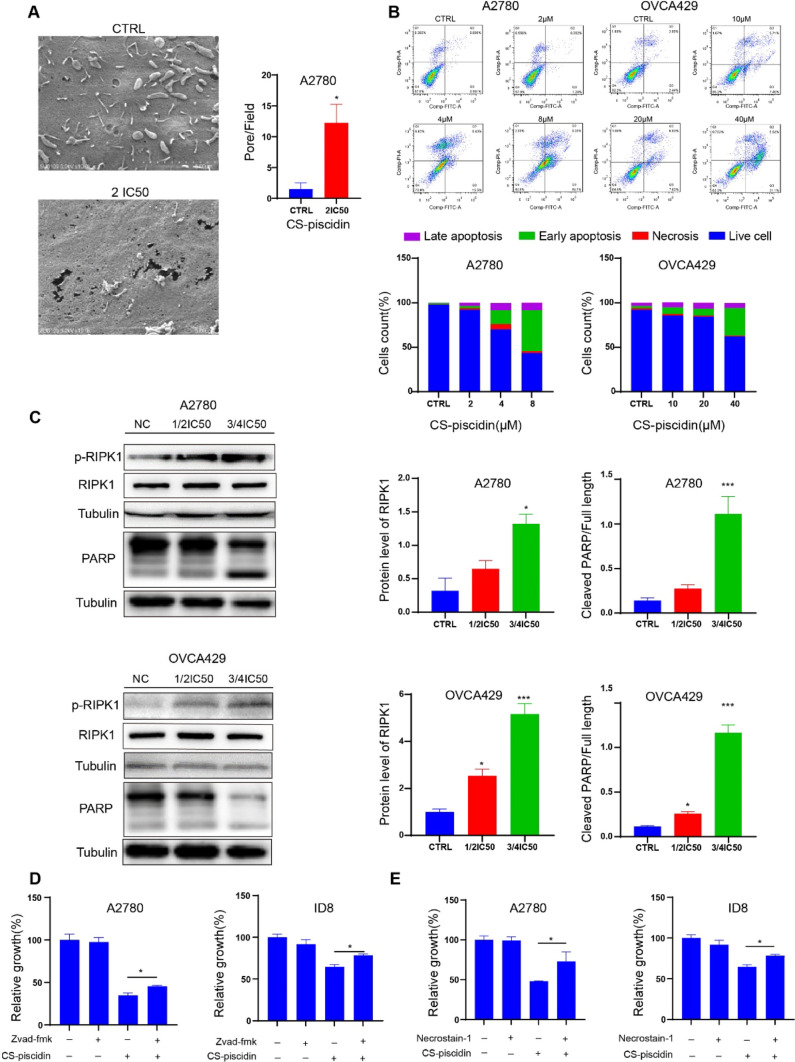
CS-piscidin activates RIPK1 and induces apoptosis. **A** The cell membrane integrity of A2780 cells after treatment with CS-piscidin was examined by scanning electron microscopy. **B** Annexin V-FITC/PI staining and flow cytometry analysis were used to quantify cell death induced by CS-piscidin in A2780 and OVCA429 cells. The results were presented as stacked bars. **C** Western blot analysis was performed to assess the alteration of p-RIPK1, RIPK1 and PARP cleavage in A2780 and OVCA429 cells after CS-piscidin treatment for 24 h. **D**, **E** The effect of CS-piscidin on cell viability was measured by treating A2780 and ID8 cells with CS-piscidin (IC50) with or without the indicated inhibitors (zVAD-FMK, 10 μM; Necrostain-1, 5 μM) for 24 h. Each experiment was performed in triplicate. Statistical significance was represented as *p < 0.05, **p < 0.01, ***p < 0.001 compared to the control group

After treatment with CS-piscidin, we observed significant cell death, which is consistent with the membrane pore-forming ability of amphiphilic peptides such as piscidin. To assess the effect of CS-piscidin on A2780 cell membrane integrity, we observed the cells under scanning electron microscopy and observed that the cell membrane was severely damaged (Fig. 2A). We also investigated whether CS-piscidin induces apoptosis and found that it significantly increased apoptosis in A2780 and OVCA429 cells, as shown by Annexin V-FITC/PI apoptosis staining (Fig. 2B up and down). RIPK1 is a sensor of apoptosis and necroptosis that can activate RIPK3 to induce cell necroptosis and can also induce apoptosis when caspase 8 is lost, resulting in PARP cleavage and apoptosis. To further investigate the mechanism of cell death induced by CS-piscidin, we checked for PARP cleavage (apoptosis) and RIPK1 phosphorylation (necroptosis) by immune blot. Our results showed that CS-piscidin activates RIPK1 by increasing its phosphorylation without changing the total RIPK1 level but enhances the cleavage of PARP (Fig. 2C). To determine the role of apoptosis and necroptosis in the cell death induced by CS-piscidin, we used the apoptosis inhibitor zVAD-FMK and the RIPK1 inhibitor necrostatin-1. Our data showed that cell death induced by CS-piscidin can be rescued by zVAD-FMK or necrostatin-1 (Fig. 2D–E).

### Myristoylated CS-piscidin improves antitumor ability and reduces cytotoxicity

**Fig. 3:**
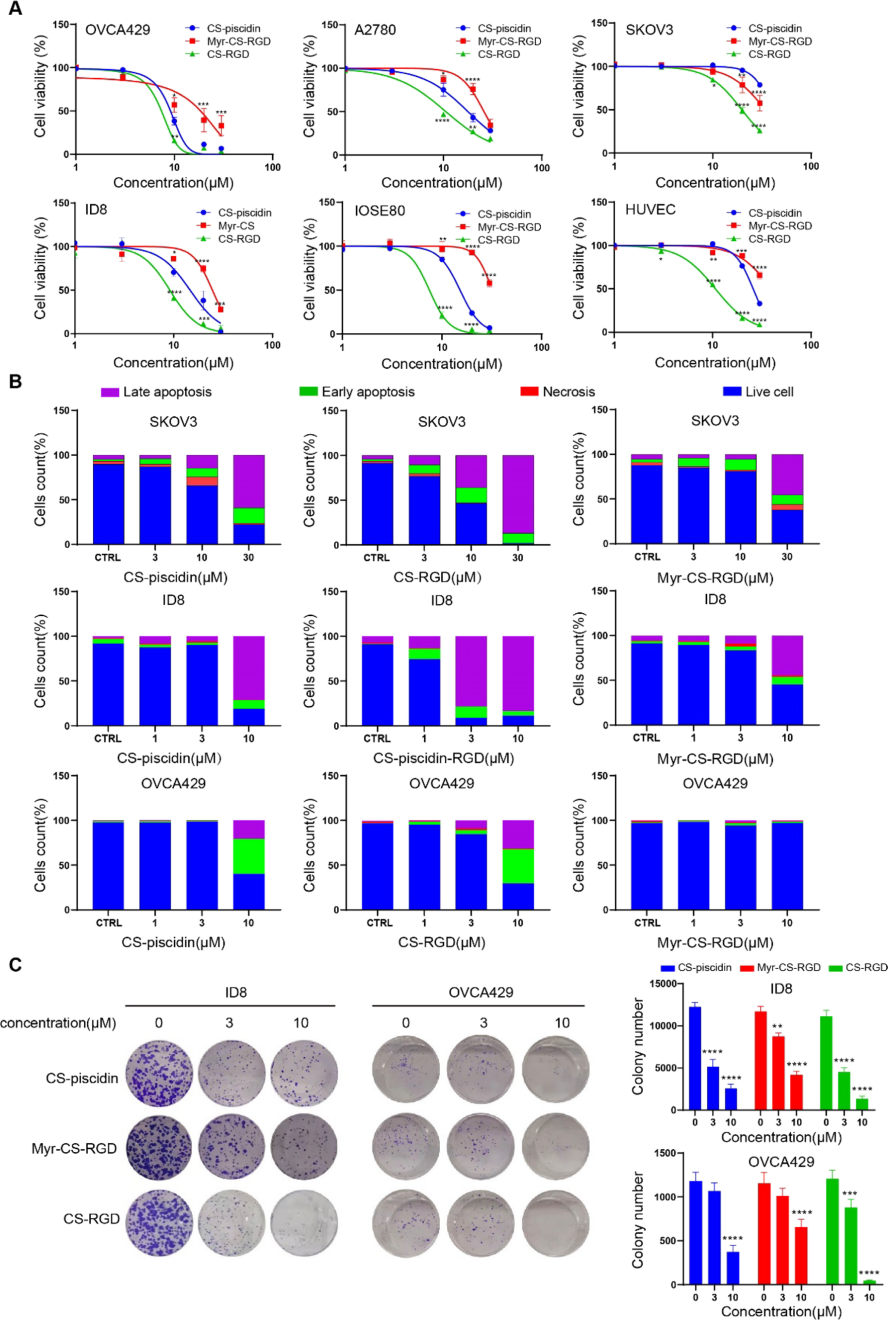
Modification of CS-piscidin improves the antitumor activity in vitro. **A** Cell viability of indicated ovarian cancer cells and normal cells treated with CS-piscidin, CS-RGD, and Myr-CS-RGD for 48 h was determined using a CCK-8 assay. **B** Annexin V-FITC/PI staining was used to analyze the ability of CS-piscidin, CS-RGD, and Myr-CS-RGD to induce cell death in SKOV3, ID8, and OVCA429 cells. The results were quantified and presented as stacked bars. **C** The long-term effects of CS-piscidin, CS-RGD, and Myr-CS-RGD on ovarian cancer cell tumorigenesis were measured by spheroid formation assay in ID8 and OVCA429 cells. All data were obtained from at least three independent experiments. **p < 0.01, ***p < 0.001 represent statistically significant differences compared with the control group

To reduce the cytotoxicity of CS-piscidin and increase its specificity, we modified it by adding the tumor-targeting peptide Cyclo-RGDfk to the C-terminus of CS-piscidin, resulting in CS-RGD. We also added a myristate group to the N-terminus of the CS-RGD peptide to enhance tumor targeting, based on previous reports indicating that cancer cells require myristic acid for cell membrane formation and energy production [23, 24]. As shown in Fig. 3A, CS-RGD exhibited stronger anticancer activity than CS-piscidin, but unexpectedly CS-RGD cytotoxicity also dramatically enhanced in normal IOSE80 cells and HUVEC cells. We checked the expression of integrin β3 level in each cell line and result showed that integrin β3 in IOSE80 cell and HUVEC cells are higher than tested ovarian cancer cell lines (Original western blot-supplementary Fig. 1). The Myr-CS-RGD has weaker anticancer activity compared with CS-piscidin in the tested ovarian cancer cell except for SKOV3 cell line, which is integrin β3 low expression cell line and insensitive to CS-piscidin and CS-RGD. Interestingly, compared to CS-RGD, Myr-CS-RGD exhibited reduced antitumor activity in most ovarian cancer cell lines (with IC50 values for SKOV3, OVCA429, A2780, and ID8 being reduced by 1.73, 2.50, 2.44, and 2.69 folds, respectively). However, Myr-CS-RGD showed more significantly reduced cytotoxicity on normal IOSE80 cells (IC50 reduced by 4.21folds) and HUVEC cells (IC50 reduced by 3.70 folds) than tumor cell lines. To confirm that the rate of apoptotic cell induction by CS-piscidin and its modified peptide, Annexin V/FITC staining was employed and the result was similar to the CCK8 results (Fig. 3B). As the CCK8 and apoptosis experiments were set for 24 h, we wondered whether myristoylation reduced the peptide's hydrophilic property and delayed its diffusion into cancer cells. Therefore, we further employed colony formation and spheroid formation assays (for 7 days) to confirm the long-term inhibition efficacy of these peptides. Our results verified that Myr-CS-RGD significantly inhibited colony and spheroid formation, which was comparable to that of CS-RGD or CS-piscidin (Fig. 3C).

### Myristylation of CS-piscidin improves antitumor efficacy in mouse ovarian cancer syngeneic model

**Fig. 4:**
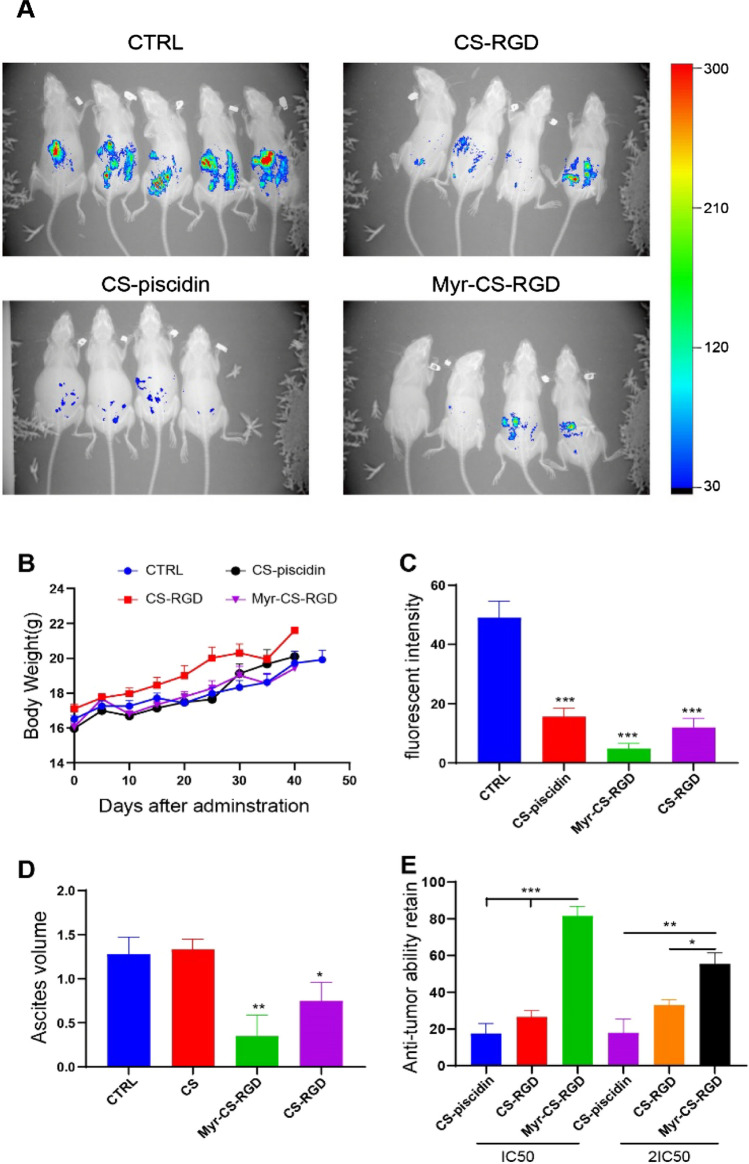
Modification of CS-piscidin improves the antitumor activity in vivo. **A** In vivo imaging was used to monitor the spread of ID8-Luc cells in mice after intraperitoneal inoculation. **B** The change in body weight was monitored since the first administration of peptides. **C** Fluorescence intensity was used to quantify the growth of ID8 cells inside the mice using Bruker MISE software. **D** The volume of ascites produced in each group was measured after the mice were sacrificed. Each group consisted of four mice in every experiment and two independent experiments were performed. **E** Peptides were pre-incubated in culture medium for 6 h and their retained antitumor ability was evaluated by CCK8 assay. *p < 0.05, **p < 0.01, and ***p < 0.001 indicate statistically significant differences compared with the control group

We further investigated the anti-cancer effects of CS-piscidin and its modified peptide in vivo using a mouse model of ovarian cancer peritoneal cavity spread. ID8-Luc cells were injected into the abdomen of mice to simulate ovarian cancer metastasis, and CS-piscidin, Myr-CS-RGD, and CS-RGD were administered intraperitoneally every other day for a total of 10 administrations at a dose of 5 mpk. All three peptides were found to inhibit tumor growth, with Myr-CS-RGD demonstrating the greatest anti-cancer activity in vivo (Fig. 4A, C). Administration of these peptides did not significantly affect the body weight of the mice (Fig. 4B). Since in vitro experiments indicated that Myr-CS-RGD had a similar long-term effect to CS-piscidin, we proposed that myristoylation may reduce the peptide's hydrophilicity, preventing its degradation. Therefore, we pre-incubated the peptide in culture medium for 6 h before using it for cell treatment. Our results showed that after 6 h of incubation, Myr-CS-RGD retained higher anti-tumor activity at two tested concentrations (IC50 and 2xIC50) than CS-RGD and CS-piscidin (Fig. 4E). Interestingly, although administration of CS-RGD and CS-piscidin reduced tumor spreading in vivo, they still produced ascites, indicating cytotoxicity to normal cells. In contrast, Myr-CS-RGD showed reduced cytotoxicity to normal cells in vivo (Fig. 4D).

## Discussion

Ovarian cancer is a critical global public health issue, with more than 125,000 women succumbing to this disease annually. It is projected that this figure will rise to 250,000 by 2035 [25]. Platinum-based combination chemotherapy is currently the preferred treatment option for ovarian cancer after surgery. However, small molecule drugs often face two major obstacles—drug resistance and side effects [26]. Therefore, the development of novel anti-OC agents with low toxicity is urgent to prevent drug resistance and tumor recurrence.

Antitumor peptides have drawn increasing attention due to their cost-effectiveness, ease of modification, and ability to overcome drug resistance. In this study, we found that combining CS-piscidin with carboplatin enhances carboplatin sensitivity in ovarian cancer cells, suggesting that a combination of CS-piscidin could reduce carboplatin resistance and avoid high dosage-induced cytotoxicity to normal cells. However, peptides have the disadvantage of a short half-life. To overcome this, peptide modification methods such as fatty acid modification and peptide cyclization have been used. Myristic acid, a 14:C saturated fatty acid that is enriched in tumor cell membrane phospholipids, was used in our study to modify the peptide and improve its stability [27]. MC-DA7R is a tumor-homing peptide modified with myristic acid by coupling it with DA7R. This modification enhances its blood–brain barrier penetrability and improves its glioma targeting ability in both in vivo and in vitro studies [13]*.* Similarly, TP5-MA was obtained by conjugating Myristic acid to a lysine-permissive site of thymopentin. This modification significantly improved its pharmacokinetic and pharmacodynamic characteristics by increasing its albumin binding affinity, thus increasing its stability in human plasma. [14]. Our results showed that short-term exposure to Myr-CS-RGD exerted a lower antitumor ability, but long-term treatment can enhance the antitumor effect, which is comparable to CS-piscidin and CS-RGD. This data suggests that myristylation of the peptide could prolong its stability by reducing its hydrophilicity, which helps resist digestion.

The RGD motif can bind to integrin αvβ3, which is typically overexpressed in cancer cells, to enhance cell–cell and cell-extracellular matrix interactions [28, 29]. We found that RGD modification enhances peptide antitumor effects but unexpectedly also increases cytotoxicity to normal cells in vitro. We tested the αvβ3 level expression in all the tested cell line and result showed αvβ3 is not specifically high expressed in ovarian cancer cell line but also significantly expressed in normal cell. Moreover, although all three peptides were able to suppress cancer cell growth in vivo, CS-RGD and CS-piscidin administration caused more ascites than Myr-CS-RGD, indicating that CS-piscidin and CS-RGD retained toxicity to normal organs leading to ascites accumulation. Our results suggest that different types of cancer exhibit distinct expression patterns of αvβ3, and therefore, solely relying on RGD modification may not be a safe strategy for anti-tumor peptide modification. However, myristylation can be an effective strategy to reduce peptide cytotoxicity and enhance tumor-targeting efficacy.

Apoptosis is regulated by two apoptotic signaling pathways: the extrinsic and intrinsic pathways [30]. The activation of the intrinsic pathway depends on the balance of BCL-2 family members [31, 32], which affect mitochondrial membrane integrity and the release of the apoptosis inducer cytochrome C [33]. RIPK1 (Receptor-Interacting Protein Kinase 1) is a protein involved in both extrinsic apoptosis and necroptosis signaling pathways. In extrinsic apoptosis, RIPK1 is a key protein that is recruited to the cytoplasmic domain of death receptors (such as FAS or TNFR1) upon ligand binding, forming a complex known as the death-inducing signaling complex (DISC) [34]. In both cases, activation of these pathways leads to caspases activation and subsequent cleavage of PARP [35]. Necroptosis is a pro-inflammatory cell death process driven by the activation of RIPK1/RIPK3/MLKL necroptosis signaling pathway, leading to cell membrane rupture and necroptosis [36]. Anticancer peptides (ACPs) typically kill cells by perforating the cell membrane and causing necrosis [37]. Interestingly, we found that CS-piscidin-induced cell death could be rescued by caspase inhibitor (zVAD-fmk) and RIPK1 inhibitor (Necrostatin-1), suggesting that CS-piscidin activates RIPK1 and induces apoptosis. It is well-known that cancer cells often escape apoptosis, which can lead to drug resistance. Therefore, simultaneous activation of both apoptosis and necrosis pathways could make peptides more efficient in killing cancer cells.

In summary, our study demonstrated that CS-piscidin has anti-ovarian cancer effects both in vitro and in vivo. The RIPK1/PARP pathway plays a critical role in mediating CS-piscidin-induced ovarian cancer cell death. Our findings also suggest that myristylation modification can improve the tumor-targeting ability and stability of the peptide. Therefore, both CS-piscidin and its modified forms have the potential to be developed as clinical treatments for ovarian cancer.

## Supplementary Information

Additional file1 (PPTX 8823 KB)

## Data Availability

The authors confirm that the data supporting the findings of this study are available within the article.
