# Supplementary material for: Antimicrobial peptides CS-piscidin-induced cell death involves activation of RIPK1/PARP, and modification with myristic acid enhances its stability and tumor-targeting capability
Source: Discov Oncol. 2023 Mar 31;14:38. doi: 10.1007/s12672-023-00642-1 (PMC10066050; doi:10.1007/s12672-023-00642-1)
Supplement: Supplementary file 1 — Additional file1 (PPTX 8823 KB) [file 12672_2023_642_MOESM1_ESM.pptx]

## Slide 1
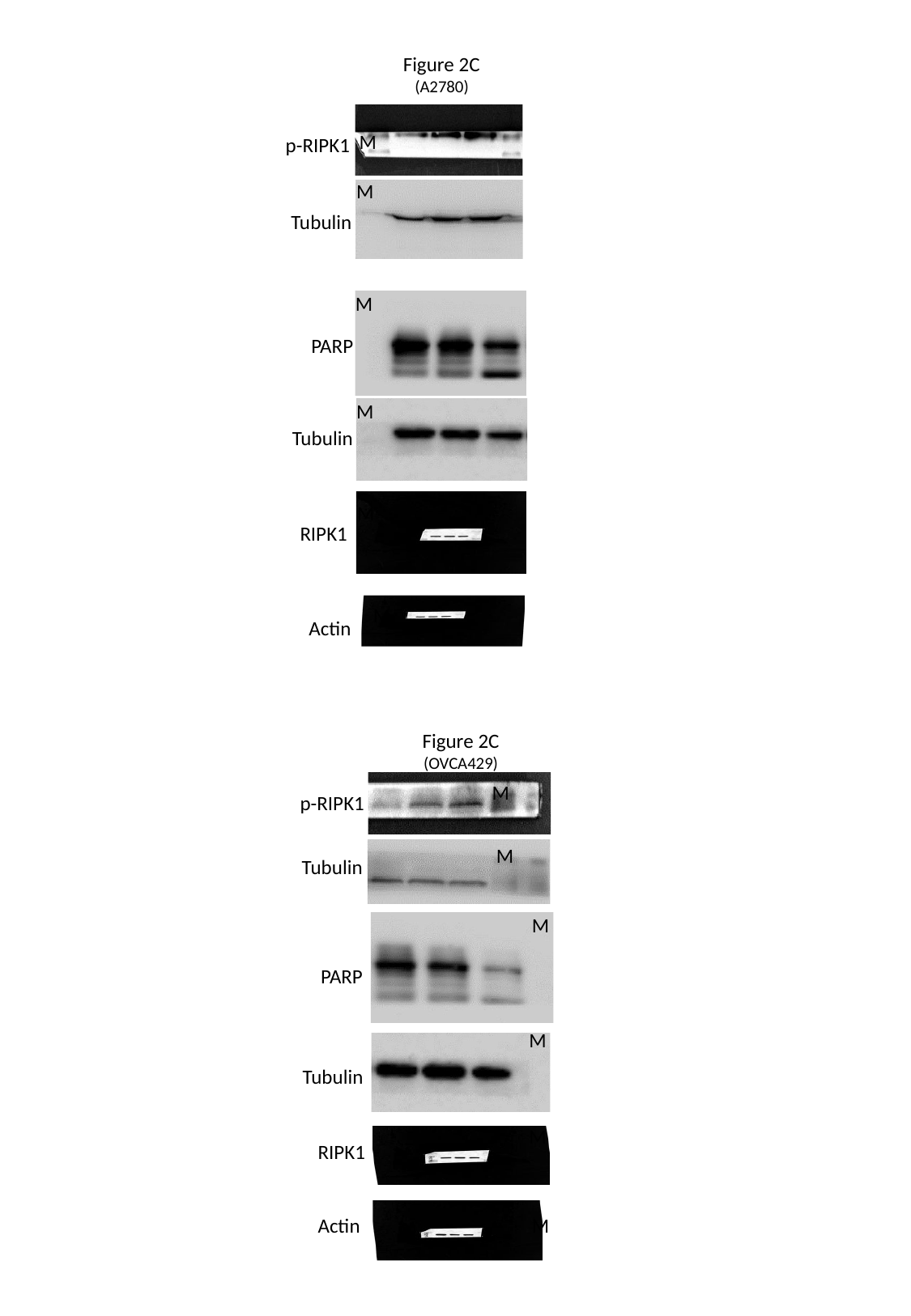

Figure 2C
(A2780)
M
p-RIPK1
M
Tubulin
M
PARP
M
Tubulin
M
RIPK1
M
Actin
Figure 2C
(OVCA429)
M
p-RIPK1
M
Tubulin
M
 PARP
M
Tubulin
M
RIPK1
Actin
M

## Slide 2
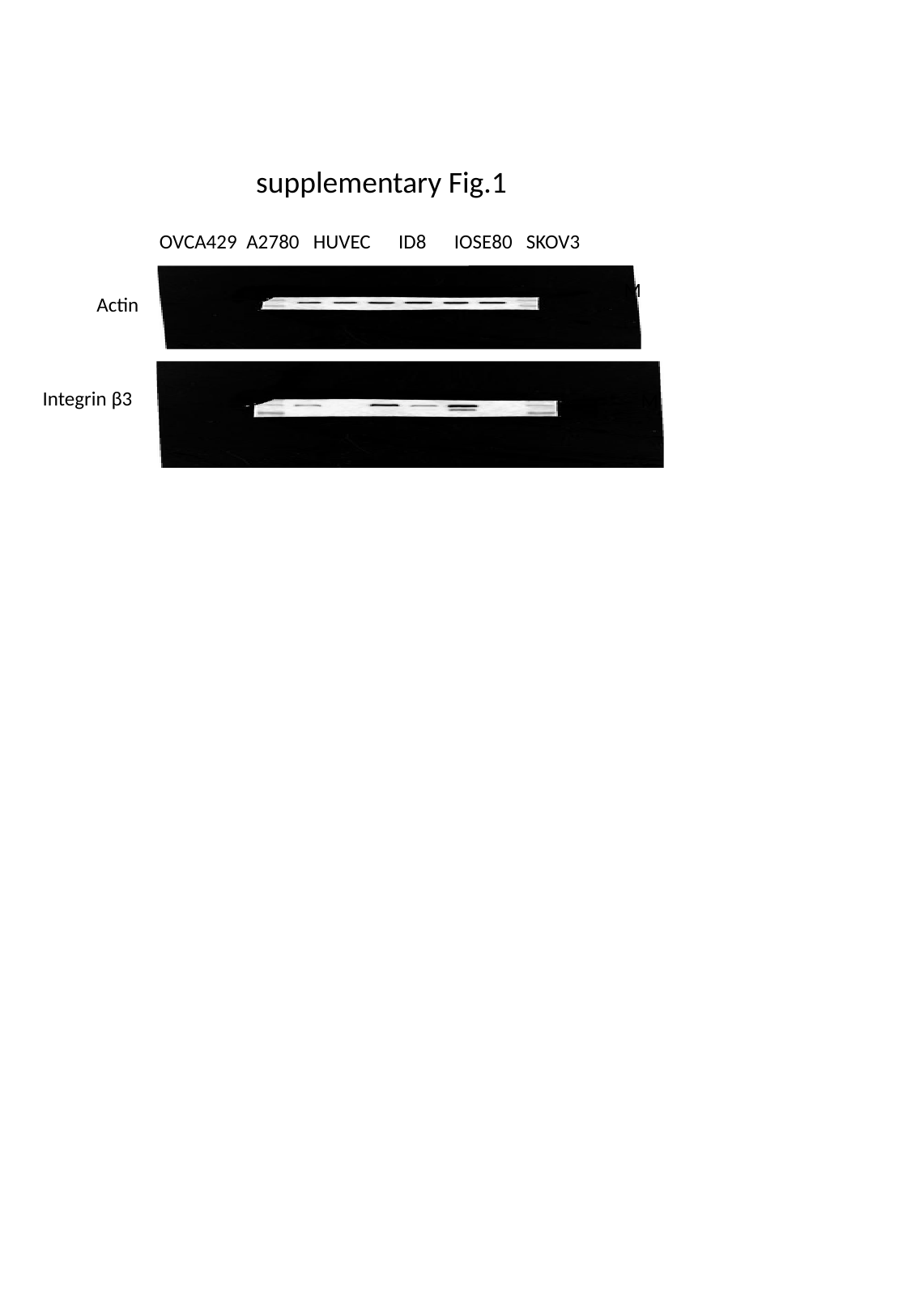

supplementary Fig.1
OVCA429 A2780 HUVEC ID8 IOSE80 SKOV3
M
Actin
Integrin β3
M
